# Association of serum cortisol level with severity of depression and improvement in newly diagnosed patients with major depressive disorder in Jimma medical center, Southwest Ethiopia

**DOI:** 10.1371/journal.pone.0240668

**Published:** 2020-10-16

**Authors:** Arefayne Alenko, Yohannes Markos, Chaltu Fikru, Eyasu Tadesse, Lealem Gedefaw

**Affiliations:** 1 Department of Psychiatry, Jimma University, Institute of Health, Faculty of Medical Science, Jimma, Ethiopia; 2 Department of Biomedical Sciences, Jimma University, Institute of Health, Faculty of Medical Science, Jimma, Ethiopia; 3 Department of Epidemiology, Jimma University, Institute of Health, Faculty of Public Health, Jimma, Ethiopia; 4 Department of Laboratory Sciences, Jimma University, Institute of Health, Faculty of Medical Science, Jimma, Ethiopia; National Institutes of Health, UNITED STATES

## Abstract

**Background:**

Major Depressive Disorder (MDD) is the leading psychiatric disorder in low- and middle-income countries, and is to be the second leading cause of burden of disease by 2020. Cortisol plays a significant role in pathophysiology of MDD. Depression can alter serum cortisol level. However, the change in serum cortisol level and its association with depressive symptom severity and improvement among patients with MDD is not well studied.

**Objective:**

To outline change in serum cortisol levels and its association with severity and improvement of depressive symptoms in newly diagnosed patients with MDD.

**Method:**

Hospital based longitudinal study was conducted among 34 newly diagnosed patients who met DSM-V criteria of MDD. Venous blood sample was performed twice; pre- and post- 8 weeks of treatment. Serum cortisol concentration was measured using an extracted radioimmunoassay. The 17-item Hamilton Depression Scale (HAM-D) was used to rate depression at baseline and after 8 weeks of treatment. Paired t-test was done to look the mean difference of serum cortisol level and HAM-D, before and after treatment. Pearson correlation was done to look the association between serum cortisol levels, HAM-D scores and, sociodemographic and clinical factors. Statistical significance was set at p<0.05.

**Results:**

There is no significant difference in cortisol concentrations at baseline and end line (t (33) = 2.02, p = 0.052). However, there is significant difference in HAM-D total score (t (33) = 5.67, p<0.001). Baseline and end line serum cortisol levels were significantly correlated (r = .561, p = .001). Monthly family income is correlated with baseline HAM-D total score (r = -0.373, p = .030). There is no significant relationship between baseline serum cortisol level and HAM-D score. There is also no significant relationship between end line serum cortisol level and HAM-D score.

**Conclusions:**

The symptoms of MDD were reduced following treatment but there is no significant difference in serum cortisol levels. Baseline and end line serum cortisol levels were significantly correlated. We recommend further research based on large sample.

## Introduction

Major Depressive Disorder (MDD) is characterized by persistent low mood and/or loss of interest in pleasurable activities which result in an inability to carry out daily activities [1]. According to World Health Organization(WHO) estimate, people living with depression increased by more than 18% between 2005–2015. More than 80% of this disease burden is among people in low- and middle-income countries (LMIC) [2].

Cortisol (17-hydroxyl-11-dehydrocorticosterone) is one of hypothalamus pituitary adrenal (HPA) axis hormone secreted from adrenal gland in response to stress [3]. A major focus in studies of the relationship between stress and depression is the role of the HPA axis, both as a marker of stress responses and as a mediator of additional pathological consequences [4,5]. It is well described that the functional changes of the HPA axis occur in patients with MDD and associated variation of serum cortisol level [5]. In contrary, presence of severe depressive symptoms is due to the high level of serum cortisol and recommended as a biomarker of MDD [6].

The association between stress and MDD is well explored. Stressful life events often precede the onset of a depressive episode. Up to 80% of depressed individuals experienced a major life event in 3–6 months prior to the onset of the depressive episode [7]. Individuals with MDD shows altered psychological and neurobiological responses to stressful life events [8,9]. As well, alterations within the stress system (HPA axis), in individuals with MDD have been studied so far [10].

Antidepressant treatment in depressed patients reduced salivary cortisol within three weeks. In addition, the reduction of salivary cortisol level has significant association with improvement of depressive symptoms [11]. In other way, increased serum level of cortisol is strongly associated with severity of MDD in treatment naïve individuals [6,12]. The serum level of cortisol before and after treatment in implies its importance to determine antidepressant treatment outcome in patients with MDD [13]. Specifically, selective serotonin reuptake inhibitors (SSRIs) treatment reduces the salivary cortisol level in women with MDD [14]. Lower awakening response of salivary cortisol was associated with an unfavorable course. There is also no association between evening cortisol level and an unfavorable course [15]. In individuals with untreated MDD, there is significant increase in plasma level of cortisol compared to healthy individuals [6,11,15]. In addition, patients with recurrent MDD have an increased level of morning serum cortisol but poor pro-social coping strategies, particularly the social joining type [16]. Cortisol can also help us to distinguish MDD from transient depressive state (TDS) in early stage of geriatric MDD [17]. In contrary, longitudinal study conducted among MDD patients shows, no difference in serum oxytocin and cortisol concentration before and after 12 weeks of SSRIs treatment [18].

The diagnosis of major depressive disorder is primarily based on subjective symptoms/manifestations according to diagnostic and statistical manual of mental disorders (DSM5), which are always assess clinically [1]. Compared with some non-psychiatric disorders, our accuracy of estimating diagnosis and treatment outcome is limited [19], which might be due to contradictory report of studies in diagnostic serum markers such as cortisol.

To date, several serum and salivary cortisol level have been studied in developed countries among patients with MDD [6,11,14,15,17,18,20]. These studies primarily focus on patients with prior history of depression not newly diagnosed individuals with MDD. In addition, these studies compared the serum cortisol level between normal individuals and individuals with mental illness. Moreover, previous studies included known patients with major depressive disorder (already started medication) and patients with multiple episodes of depression, even though the findings are contradicting [15,18]. The linear relationship between serum cortisol level and severity of depressive symptoms were not taken account in previous studies. Since there is paucity of study in sub-Saharan African, especially regarding serum cortisol level and depressive symptom severity, this study has clinical significance and can serve as baseline data for further studies.

In sub-Saharan countries, there is stress related to social and political instability, and low living standard. These stressful phenomena can alter serum cortisol in addition to depression. However, there is limited investigation done in sub-Saharan countries. Therefore, this study investigated the association of serum cortisol level with depressive symptom severity and improvement in patients with newly diagnosed MDD in sub-Saharan countries.

## Methods and materials

### Study area and design

Hospital based longitudinal study was conducted in Jimma medical center (JUMC) psychiatric clinic. JUMC is one of the oldest public hospitals in Ethiopia. Psychiatric clinic in JUMC is giving services for mentally ill patients in inpatient department (IPD) and outpatient department (OPD) for about 15 million people in southwest Ethiopia. Currently, there are around 1000 patients who are attending follow up treatments at OPD monthly and on average 70 patients are visiting daily. There are about 65 beds in IPD. The study was conducted from March to August, 2019.

### Study population and sampling technique

Patients who met DSM-V criteria of MDD [1] and were planned to attend follow up treatment at Jimma medical center psychiatric clinic were included in the study. Among Patients’ with MDD, newly diagnosed were included in the study. The following patients were excluded from the study.

- Patients had been receiving antidepressants, benzodiazepines, mood stabilizers and anti-epileptic medications.
- Individuals with previously diagnosed MDD and taking treatment, multiple episodes of depression and treatment resistant cases.
- Patients who used herbal medication 2 weeks prior to the study were excluded.
- Patients with comorbid psychiatric and physical illness.
- Females taking hormone-based contraceptives and postmenopausal women.

Patients’ selection was aimed to minimize psychiatric co-morbidity and avoid miss diagnosis. Patients were initially screened at OPD by BSc psychiatry professionals during their first visit, and then the diagnosis of MDD was confirmed by mental health specialists based on DSM-V MDD criteria.

We used consecutive sampling technique to recruit 34 study participants based on inclusion criteria among patients visiting Jimma University medical center psychiatric clinic from March 01 to June 30, 2019.

### Data collection tool and procedure

A 17-item Hamilton Depression Scale (HAM-D) [21] was used to assess severity of depression at baseline and monitor response after 8 weeks of treatment. DSM-V diagnostic criteria was used to assess and exclude anxiety disorder, schizophrenia spectrum and other psychotic disorder, bipolar and related disorders, and substance use disorders. Patients were eligible for inclusion if they fulfill diagnostic criteria for MDD based on DSM-V criteria and were further assessed as having MDD as the primary illness at psychiatric interview by mental health specialist. A structured and interviewer administered questionnaire was used to collect socio-demographic data and, clinical and help seeking pattern of patients. Six BSc psychiatry professionals and one mental health specialist were conducted data collection.

### Sample collection procedure

#### Baseline assessment of medical illness

Before confirming the psychiatric diagnosis of MDD, other medical (physical) illness were assessed through baseline laboratory investigation of complete blood count, thyroid function test, liver function test, renal function test, fasting blood sugar and erythrocyte sedimentation rate (ESR). Vital sign of patients’ were checked for possible derangement.

#### Baseline cortisol

Within 1week of confirming diagnosis of MDD, initial venous blood sample of 4ml was taken. Patients then started treatment with fluoxetine and amitriptyline which was determined on clinical grounds in consult with a treating clinician. No structured psychotherapy was delivered to the patients in the context of the research study, or external to it. Psycho-education was provided for care givers.

#### End line cortisol (post treatment)

The second venous blood sample was taken after 8 weeks of taking antidepressant treatment. Patients were examined every week for the purposes of the study and/or more frequently if required on clinical grounds.

For each patient, both baseline and end line samples were collected at the same time of day (4:00–5:00pm) afternoon. For all participants, 24 hours preceding study visits; coffee, alcohol, tobacco, smoking and khat (local stimulant plant) were prohibited. The sample was collected by diploma nurses and standard operating procedure of nursing practice is strictly followed during sample collection. The collected sample was immediately sent to laboratory for analysis.

### Cortisol Radioimmunoassay (RIA)

All samples were measured in a single assay. Serum cortisol concentrations were measured before and after 8 weeks of antidepressant treatment using an extracted RIA. RIA used hydrocortisone (*H-4001*, *Sigma Chemical Company*, *St Louis*, *MO*, *USA*) as a standard. The assay utilized ^3^H-cortisol (*Amersham Pharmacia Biotech UK*, *Buckinghamshire HP*, *England*) as tracer and a dichloromethane extraction procedure with a mean (± SEM) recovery of 91.2 ± 1.8%. All samples were measured in a single assay, which had a sensitivity of 0.54 ng/ml. The cross reactivity to chemicals that interfere with the assay result and their concentration (ug/dL) was checked. The cross reactivity of chemicals were: 11-Deoxycortisol (13.6%), cortisone (1.4%), dexamethasone (2.0%), corticosterone (8.0%), and prednisolone (32.2%).

### Data management and statistical analysis

Data is double entered to Epi data software version 4.1 to minimize error. Then it is exported to IBM SPSS version 24 for analysis. Descriptive statistics (frequency count, percentage, Median, and standard error) were computed for continuous and/or categorical variables. Paired (dependent) t-test was run to look the mean difference of serum cortisol level and HAM-D score, before and after antidepressant treatment. Pearson correlation was done look the association between change in serum cortisol, change in HAM-D score and, sociodemographic and clinical factors. Variables were included in Pearson correlation analysis after checking assumptions. A significance level of 5% (two sided) was used to declare statistical significance (p<0.05).

### Ethics approval and consent to participate

Ethical approval was obtained from the Institutional Review Board of Jimma University Institute of Health **(Ref. No: IHRPGD/662/2019)**. Patients who agreed to participate gave written informed consent. Confidentiality was maintained by omitting identifiers from study tool and privacy was ensured during the interview. All participants were given an information sheet and only included in the study after providing informed written consent.

## Results

### Sociodemographic characteristics and clinical factors of study participants

The study included 34 participants with newly diagnosed MDD (17 females, 17 males) ranging in age from 24 to 45 year (Mean ±SD, 33.26 ± 5.96 year). Among study participants, 15 (44.1%) were in the age range of 31–37 year. Majority of study participants were divorced 14(44.1%) and achieved secondary education 16 (47.1%). Regarding occupation, half 17(50%) were not involved in any occupation. Majority, 22(64.7%) of study participants live in urban residents. The average monthly family income was 1272 ETB with minimum and maximum income of 500 and 4500, respectively. The average duration of illness was 13.5month with minimum and maximum duration of 5.0 and 28.0 month, respectively. Twenty three (67.6) study participants reported the cause of illness was evil spirit. Only 9(26.5%) study participants have family history of mental illness **(Table 1).**

**Table 1:** Sociodemographic characteristics and clinical factors of study participants in Jimma medical center Southwest Ethiopia, 2019. (n = 34).

Variable	Frequency	Percent
**Age of participants**		
24–30	12	35.3
31–37	15	44.1
38–45	7	20.6
**Marital status**		
Married	9	26.5
Single	9	26.5
Divorced	14	41.1
Widowed	2	5.9
**Educational status**		
No formal education	4	11.8
Primary education	10	29.4
Secondary education	16	47.1
Higher education	4	11.8
**Occupation**		
Farmer	5	14.7
no formal occupation	18	53.0
Government worker	5	14.7
Student	6	17.6
**Residence**		
Urban	22	64.7
Rural	12	35.3
**Perceived cause of illness**		
Evil spirit and witchcraft	23	67.6
Stressful life event	9	26.4
Difficult family environment	2	6.0
**Family history of mental illness**		
Yes	9	26.5
No	25	73.5

### Serum cortisol level and symptoms of major depressive disorder

**Fig 1:**
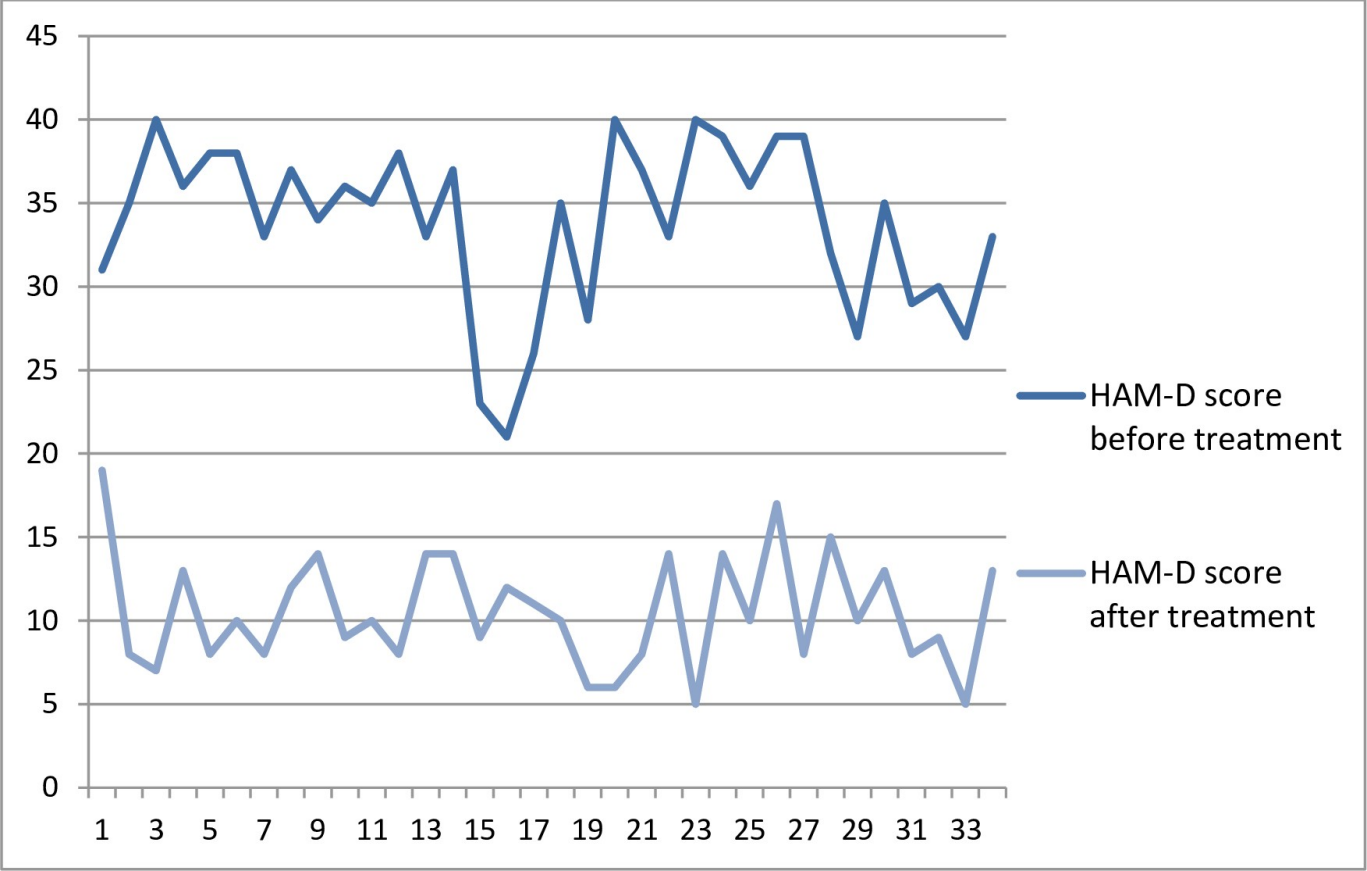
Hamilton depression rating scale (HAM-D) total score before and after antidepressant treatment among study participants in Jimma medical center psychiatric clinic, 2019.

The median with standard deviation (x±SD) serum concentrations of cortisol (ug/dL) before and following an antidepressant treatment was (8.60±1.90) and (8.50±1.56), respectively. Baseline HAM-D score shows all patients had severe depression with a mean (±SD) HAM-D score of 33.82 ± 4.98 and, maximum and minimum value of 21 and 40, respectively. All patients shown significant improvement (a decrement of ≥50% from baseline HAM-D score) after antidepressant treatment with a mean (±SD) HAM-D score of 10.50± 3.41 and, maximum and minimum value of 5 and 19, respectively **(Fig 1).**

There is no significant mean difference in serum cortisol level before and after treatment with t (33) = 2.02(95%CI, 1.148–0.004) and p = 0.052, even though there is significant difference in HAM-D total score with t (33) = 5.67(95%CI, 6.13–12.99) and p<0.001. Baseline and end line serum cortisol levels were significantly and positively correlated with r = .561 and p = .001. This corresponds to a coefficient of determination (R2) of 0.31, suggesting that about 31% of the variability of end line serum cortisol level can be explained by the relationship with baseline serum cortisol level. However, there is no significant correlation between baseline serum cortisol level and baseline HAM-D score (r = .197, p = 0.264). There is also no significant association between end line (post-treatment) serum cortisol level and HAM-D score (r = .156, p = .379). Regarding sociodemographic factors, monthly family income is negatively correlated with baseline HAM-D total score (r = -0.373, p = .030). The coefficient of determination (R2) of 0.14 tells us that 14% of the variation in the baseline HAM-D total score is reduced by taking into account the monthly income of family **(Table 2).**

**Table 2:** Pearson correlations among serum cortisol level, HAM-D total score, age, income and duration of illness in patients with newly major depressive disorder in Jimma medical center, 2019(n = 34).

	1	2	3	4	5	6	7
**1** Baseline serum cortisol level	1						
**2** Baseline HAM-D total score	.197	1					
**3** End line serum cortisol level	.561**	-.064	1				
**4** End line HAM-D total score	.234	-.137	.156	1			
**5** Age	.044	-.120	.237	.006	1		
**6** Monthly family income	-.222	-.373*	-.228	.025	.312	1	
**7** Duration of illness	.016	.088	.015	.228	.160	.019	1

**. Correlation(r) is significant at the 0.01 level (2-tailed).

*. Correlation(r) is significant at the 0.05 level (2-tailed).

**Fig 2:**
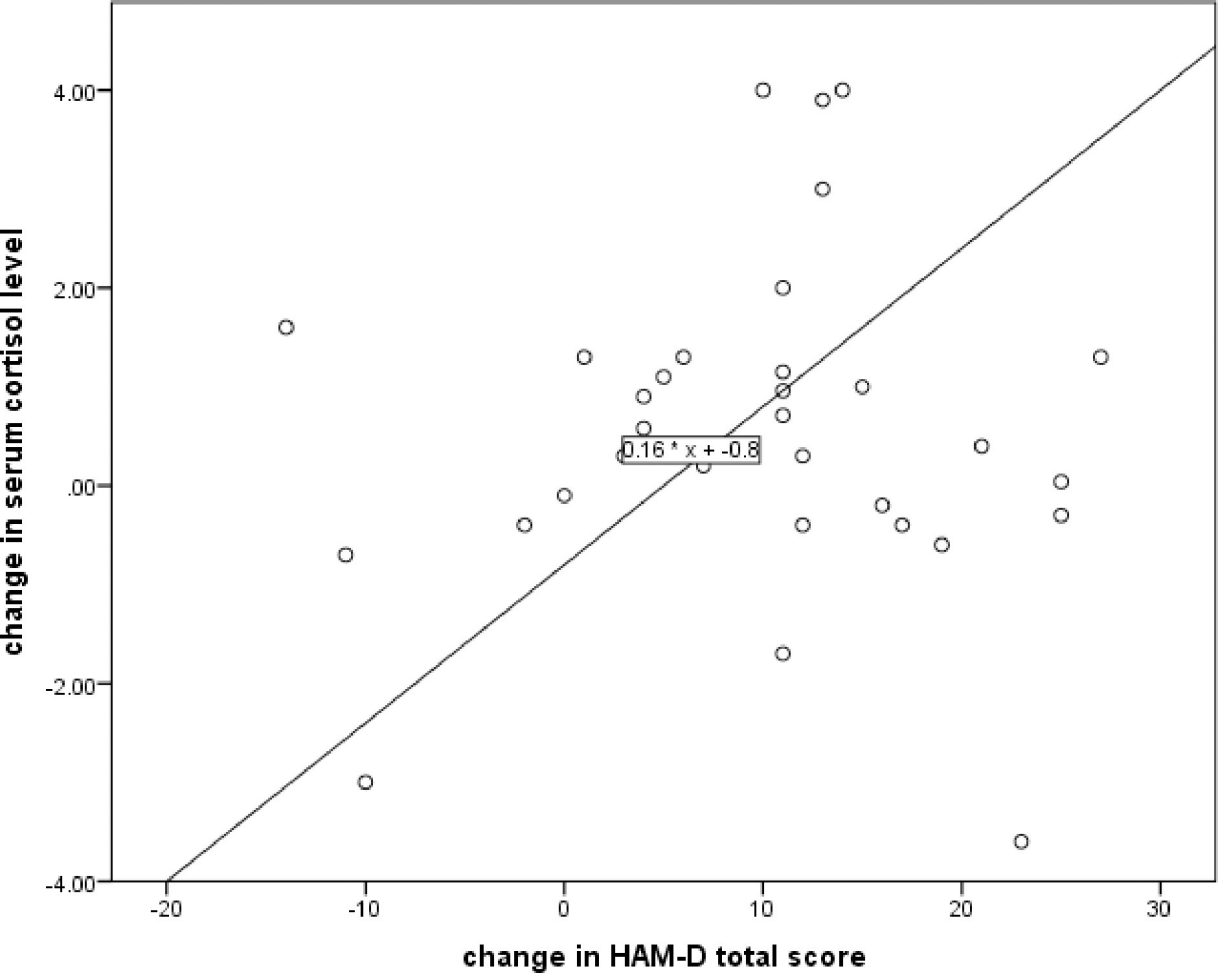
Change in serum cortisol level and change in HAM-D scores, before and after treatment, among study participants in Jimma medical center psychiatric clinic, 2019.

Even though there is significant change in HAM-D score before and after treatment, there is no correlation with changes of cortisol concentration before and after treatment (r = 0.048, p = 0787) **(Fig 2).** Change in serum cortisol level is not significantly correlated with age of participants (r = -0.173, p = 0.327) and duration of illness (r = 0.004, p = 0.984). Similarly, change in HAM-D total score is not correlated with age of participants (r = -0.066, p = 0.712) and duration of illness (r = -0.139, p = 0.439).

## Discussion

There is no significant mean change in serum cortisol level before and after treatment with t (33) = 2.02 and p = 0.052. However, baseline and end line serum cortisol levels were significantly correlated with r = .561 and p = .001. This corresponds to a coefficient of determination (R2) of 0.31, suggesting that about 31% of the variability of end line serum cortisol level can be explained by the relationship with baseline serum cortisol level. Result shows there is significant difference in HAM-D total score with t (33) = 5.67 and p<0.001. However, there is no significant correlation between baseline serum cortisol level and baseline HAM-D score (r = 0.075, p = 0.264). There is also no significant correlation between end line (post-treatment) serum cortisol level and HAM-D score (r = 0.21, p = 0.231).

Our finding indicate that cortisol serum level in patients with newly diagnosed major depressive disorder is not influenced by antidepressant treatment suggesting that depression did not significantly influence activity of the HPA axis. This outcome is in agreement with previous research that has demonstrated no change in serum cortisol concentrations following selective serotonin reuptake inhibitors (SSRIs) treatment in a sample of 16 patients with MDD, before and after 12 weeks of treatment [18]. The other study in line with our finding, reported that there is no association between evening cortisol level and an unfavorable course of depressive symptoms [22], even though the association was not well explored.

In contrary to our finding, antidepressant treatment in depressed patients reduced salivary cortisol and improve/decrease depressive symptoms within three weeks [11]. Similarly, increased serum level of cortisol is strongly associated with MDD [6]. The variation of serum level of cortisol before and after antidepressant treatment was well elaborated in studies conducted among patients with MDD [6,11,23]. Selective serotonin reuptake inhibitors (SSRIs) treatment reduces the salivary cortisol level in women with MDD [14]. The difference might be due to the fact that; while measurements of cortisol and other stress hormones are notoriously difficult to control as the time of sample collection, seasonal variation, exercise before sample collection, psychoactive substance use, medical illness and the immediate events right before sampling can all affect biological measurement of cortisol [24]. In our study, we controlled for time of sample collection, coffee intake, alcohol use and abuse; tobacco smoking and khat (local stimulant plant) intake within 24 hours of study visits, and general physical illness.

In contrary, 15 patients with recurrent major depressive disorder had increased level of morning serum cortisol compared with healthy controls [16]. Individuals with depression (compared with healthy controls) often demonstrate abnormal diurnal rhythm of cortisol with lower morning cortisol levels and higher evening levels [25]. Meta-analyses suggest, however, that MDD is associated with hypercortisolism at certain times of the day [25,26]. The difference might be due to relatively small size (n = 15), inclusion of patients with recurrent depression and the time of sample collection.

In our study, both fluoxetine and amitriptyline were effective in treating symptoms of MDD in newly diagnosed patients (at least 50% reduction in symptoms following 8 weeks treatment). Although it is well studied that serum and salivary cortisol level rise among patients with MDD [11,14,23], the mechanism is not well understood and the direction of dysregulation of HPA axis can furthermore vary. Nevertheless, studies shows sustained increment of cortisol level following antidepressant therapy has been linked to treatment resistance cases of MDD [27,28]. Therefore, selecting patients with recurrent depression or resistant to treatment (with a matched healthy control group before treatment) may help to determine how dysregulation of the HPA axis is associated with psychopathology of depressive symptoms experienced by specific patient, and whether the HPA axis may represent a significant contribution in severity and treatment outcome of MDD.

Evidence shows depression like behaviors in rodents/mice is due to effect of stress on mitochondrial energy metabolism. This evidence is proved by the effect of corticosterone treatment for 6 weeks and mice displayed depression like behaviors [29]. The other study shows corticosterone 20mg/kg subcutaneous for 6 weeks can induce depression like behavior in mice. The corticosterone (CORT) induced depression like behavior in mice was ameliorated by nicotinamide mononucleotide (NMN) 300mg/kg oral dose for 2weeks. NMN ameliorates the depression like behaviors induced by CORT through attenuation of the disruption of mitochondrial energy production [30]. In human being, deviated functioning of the HPA axis seems to be involved in depression. However, the mechanisms underlying the relationship between stress, cortisol and depressive symptoms, like mice/rodent, are not completely resolved yet. Neurotransmitters in the brain like serotonin and GABA are highly responsive to stress and depression, but this response is depend on the exact nature and severity of stressor and depression [31].

Monthly family income is negatively correlated with baseline HAM-D total score (r = -0.373, p = .030). This tells us that 14% of the variation in the baseline HAM-D total score is reduced by taking into account the monthly income of family. In other way when family income decrease/low, depressive symptom severity increases. Previous studies support our finding [32–35]. This might be due to the fact that low income is associated with stress due to lack of means to fulfill daily living. Therefore, Stress can mediate the observed association between depressive symptom severity and family income [34,36,37].

### Strength and limitation of the study

This study has the following strength: two point sample collection, inclusion of new cases, controlling possible causes of HPA axis dysfunction (physical illness, substance/drug abuse, tradition medicine use, and other psychiatric illness), excluding comorbidity and used standard laboratory procedures with less cross-reactivity.

A limitation of the current study, however, is that serum cortisol is not sampled across the day in both before and after treatment samples. Nevertheless, the fact that our results were in a small number of patients, and no differences in cortisol concentrations were detected from before relative to following treatment, evidence to the weakness of our sampling procedure. Hence, future research efforts should assess blood cortisol level across the day is recommended.

## Conclusion and recommendation

In summary, this single population longitudinal study presents finding on serum cortisol level before and after antidepressant among newly diagnosed patients of major depressive disorder. There is no significant change in serum cortisol level before and after treatment, even though there is significant depressive symptom improvement. However, baseline and end line serum cortisol levels were significantly correlated. Monthly family income is correlated with baseline HAM-D total score. In patients with MDD, emphasis must be given to family income during baseline assessment. We recommend further research using large sample.

## Supporting information

S1 File (SAV)
